# Elemental imaging shows mercury in cells of the human lateral and medial geniculate nuclei

**DOI:** 10.1371/journal.pone.0231870

**Published:** 2020-04-22

**Authors:** Roger Pamphlett, Stephen Kum Jew, Philip A. Doble, David P. Bishop

**Affiliations:** 1 Discipline of Pathology, Brain and Mind Centre, Sydney Medical School, The University of Sydney, Sydney, Australia; 2 Department of Neuropathology, Royal Prince Alfred Hospital, Sydney, Australia; 3 Elemental Bio-Imaging Facility, School of Mathematical and Physical Sciences, University of Technology Sydney, Sydney, New South Wales, Australia; Chinese Academy of Sciences, CHINA

## Abstract

**Objective:**

Interference with the transmission of sensory signals along visual and auditory pathways has been implicated in the pathogenesis of hallucinations. The relay centres for vision (the lateral geniculate nucleus) and hearing (the medial geniculate nucleus) appear to be susceptible to the uptake of circulating mercury. We therefore investigated the distribution of mercury in cells of both these geniculate nuclei.

**Materials and methods:**

Paraffin-embedded tissue sections containing the lateral geniculate nucleus were obtained from 50 adults (age range 20–104 years) who at autopsy had a variety of clinicopathological conditions, including neurological and psychiatric disorders. The medial geniculate nucleus was present in seven sections. Sections were stained for mercury using autometallography. Laser ablation-inductively coupled plasma-mass spectrometry was used to confirm the presence of mercury.

**Results:**

Ten people had mercury in cells of the lateral geniculate nucleus, and in the medial geniculate nucleus of three of these. Medical diagnoses in these individuals were: none (3), Parkinson disease (3), and one each of depression, bipolar disorder, multiple sclerosis, and mercury self-injection. Mercury was distributed in different groups of geniculate capillary endothelial cells, neurons, oligodendrocytes, and astrocytes. Mass spectrometry confirmed the presence of mercury.

**Conclusion:**

Mercury is present in different combinations of cell types in the lateral and medial geniculate nuclei in a proportion of people from varied backgrounds. This raises the possibility that mercury-induced impairment of the function of the geniculate nuclei could play a part in the genesis of visual and auditory hallucinations. Although these findings do not provide a direct link between mercury in geniculate cells and hallucinations, they suggest that further investigations into the possibility of toxicant-induced hallucinations are warranted.

## Introduction

**Fig 1:**
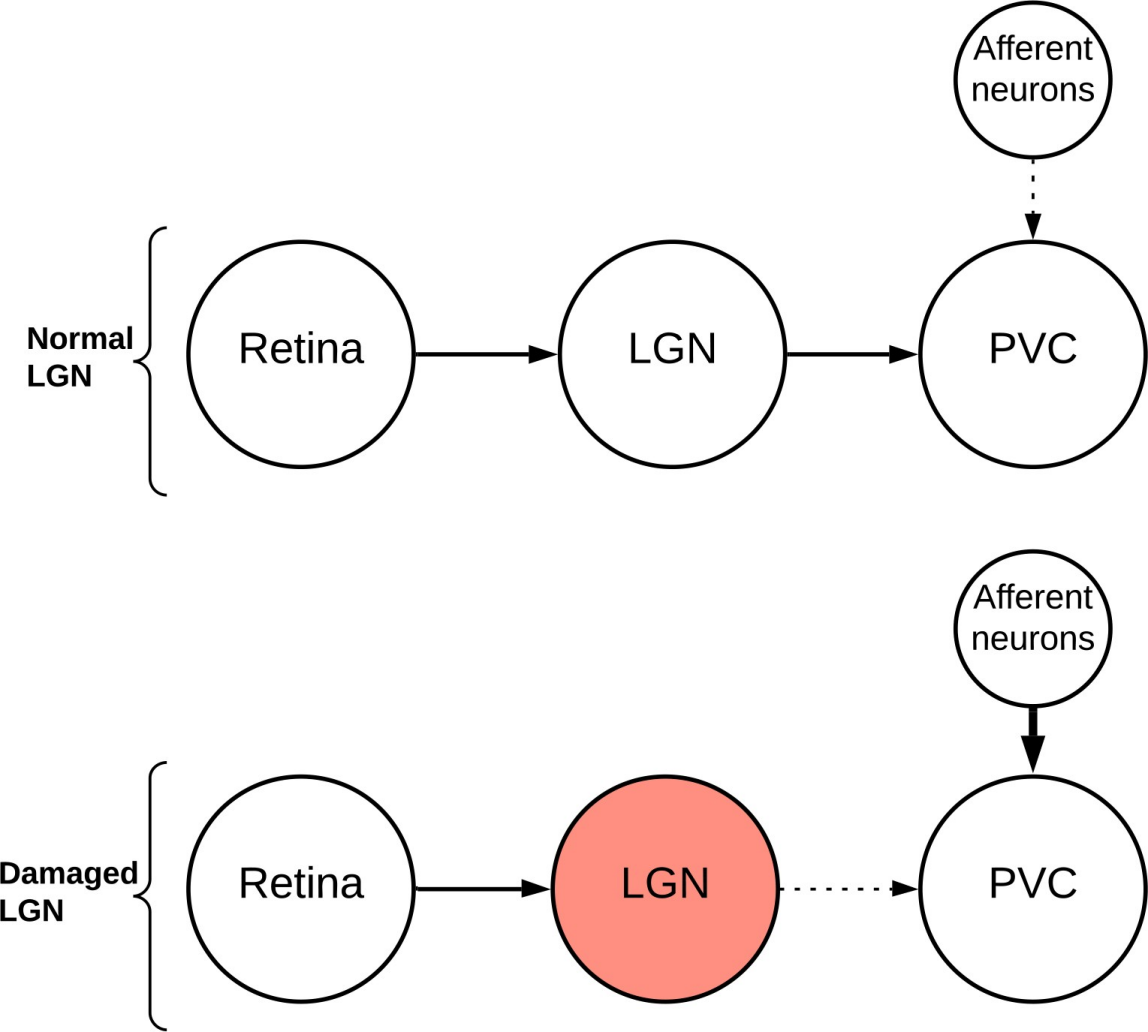
The defective information processing hypothesis for the origin of visual hallucinations. *Normal LGN*. Visual signals from the retina are filtered and reorganised by the LGN before transmission to the primary visual cortex (PVC). Other afferent neurons also synapse with neurons in the PVC. *Damaged LGN*. Neuronal transmission from the retina to the PVC is impaired after damage (red) to the LGN. Other afferent neurons to the PVC compensate by increasing their activity, stimulating the PCV to generate visual hallucinations.

Visual and auditory hallucinations, perceptions that occur in the absence of corresponding sensory stimuli, can arise from a wide range of drug-induced, medical and psychiatric conditions, as well as in the general population [1]. It has been proposed that hallucinations appear because of interference with the visual or auditory pathways, followed by defective information processing [2] (**Fig 1**). The thalamic lateral geniculate nucleus (LGN) is the main relay centre in the visual pathway, and the nearby medial geniculate nucleus (MGL) in the auditory pathway. These geniculate nuclei are therefore candidate sites for damage that could result in overactivity of the primary visual and auditory cortices. Attempts to find histopathological changes in the LGN in conditions where hallucinations are common, such as schizophrenia, have so far been unsuccessful [3,4], though magnetic resonance imaging studies suggest the LGN can be smaller than normal in Parkinson disease [5] and multiple sclerosis [6], disorders in which hallucinations have been described [7,8].

One human study, using neutron activation analysis on fresh brain tissue from 17 randomly selected autopsies, showed that, while average mercury levels were highest in the cerebellum (0.28 μg/g), the next highest levels were in the geniculate bodies (0.20 μg/g), with lower levels in other regions of the brain such as the pons and the calcarine cortex [9]. Several animal experiments suggest mercury enters the geniculate nuclei preferentially [10–16]. In monkeys exposed to methylmercury, average tissue levels of mercury were higher in the LGN than in other regions of the brain [10]. Monkeys exposed to methylmercury had ultrastructural changes in neurons of the LGN [11]. Damage to LGN neurons has been described in a mercury-exposed dog [12], and bismuth has been seen to accumulate in the MGN of mice [13]. In rats exposed to various forms of mercury, mercury was visible in the LGN and MGN, though usually only at higher levels of exposure [14–16].

Mercury in neurons can cause axonal atrophy without producing light microscopic signs of damage in neuronal cell bodies [17]. This implies that mercury could be an agent that interferes with the function of geniculate neurons, given that histological changes in the cell bodies of geniculate neurons are rarely present in humans [3,4]. We therefore investigated whether any people from varied clinicopathological backgrounds had mercury in cells of their LGN or MGN.

## Methods

### Ethics statement

This study (X14-029) was approved by the Human Research Committee, Sydney Local Health District (Royal Prince Alfred Hospital Zone), and by the Office of the New South Wales Coroner. The institutional review board waived the need for written informed consent from relatives to use the tissues removed for standard diagnostic purposes at coronial autopsy from these deceased individuals for scientific purposes since this was a de-identified retrospective study of archived paraffin-embedded tissue.

### Cases

Cases selected for study were 48 adults who had coronial autopsies performed at the Glebe Department of Forensic Medicine, Sydney, in whom (1) archived paraffin blocks of the LGN and adjacent hippocampus were available, and (2) the number of locus coeruleus neurons that contained mercury (a potential marker of previous mercury exposure) had been measured in a previous study [18]. Two adults who were donors to the Multiple Sclerosis Research Australia Brain Bank at the Brain and Mind Centre, Sydney, were also included. The 50 individuals comprised 26 males (age range 20–98 years, mean age 47 years) and 24 females (age range 29–104 years, mean age 66 years) (**S1 Table**). Pre-mortem medical conditions were: none (N = 12), Parkinson disease (N = 9), schizophrenia (N = 7), Alzheimer disease (N = 5), depression (N = 5), bipolar disorder (N = 3), Huntington disease (N = 2), multiple sclerosis (N = 2), and one each of anorexia nervosa, Lewy body disease, myotonic dystrophy, progressive supranuclear palsy, and mercury self-injection. Mercury levels in the individual who injected himself with metallic mercury were greatly raised in organs such as the kidney and liver, but only slightly higher than normal in the cerebrum [19], indicating that relatively few brain cells took up the circulating mercury. Causes of death were suicide (N = 14), cardiovascular disease (N = 10), infection (N = 8), trauma (N = 6), drowning (N = 4), drug overdose (N = 2), undetermined (N = 4), and one each of burns and undernutrition (**S1 Table**).

### Autometallography (AMG)

Paraffin blocks taken from the LGN and adjacent hippocampus were sectioned at 7 μm. In seven blocks the medial geniculate nucleus was also present. Sections were stained for inorganic mercury bound to sulphide or selenide using silver nitrate autometallography (AMG), which represents the presence of mercury as black grains [20]. AMG is a sensitive photographic technique that can detect as few as 10 mercury sulphide/selenide molecules in a cell [21]. Briefly, sections were placed in physical developer containing 50% gum arabic, citrate buffer, hydroquinone, and silver nitrate at 26°C for 80 min in the dark then washed in 5% sodium thiosulphate to remove unbound silver. Sections were counterstained with mercury-free hematoxylin or Luxol fast blue and viewed with bright-field microscopy. Other sections were stained with hematoxylin and eosin to assess general pathology, and hematoxylin alone to act as a control for the AMG/hematoxylin. In two mercury-positive LGN individuals, sections were immunostained after AMG/hematoxylin with glial fibrillary acidic protein antibody to see if mercury was present in astrocytes; briefly, sections were treated with hydrogen peroxide to block endogenous peroxidase activity, antigen retrieved by heating, non-specific antigen blocked with horse serum, rabbit anti-human glial fibrillary acidic protein polyclonal antibody (Dako M0761) applied at a 1:2000 dilution, and diaminobenzidine tetrahydrochloride used for visualisation. Each staining run included a control section of mouse spinal cord where motor neuron cell bodies contained mercury following an intraperitoneal injection of mercuric chloride [17]. AMG is a histochemical amplification technique, so it cannot be used to quantitate the amount of mercury in individual cells. However, because the timing of the reactions was kept the same between runs, different categories of staining between samples could be estimated: the category of AMG staining in groups of cells was graded as absent if no cells stained, moderate if fewer than 50% of cells stained, and marked if more than 50% of cells stained.

### Laser ablation-inductively coupled plasma-mass spectrometry (LA-ICP-MS)

In addition to mercury, AMG detects inorganic silver and bismuth [20,22]. To confirm that AMG in this tissue was due to mercury, a 7 μm paraffin section from an AMG mercury-positive case was subjected to LA-ICP-MS to qualitatively determine the presence of mercury, silver, bismuth, and phosphorus (the latter to assess cell density), as well as for aluminium, cadmium, chromium, gold, iron, lead, and nickel. Analyses were carried out on an NWR193 excimer laser (Kenelec Scientific) hyphenated to an Agilent Technologies 7700 ICP-MS fitted with ‘s’ lenses for enhanced sensitivity, with argon used as the carrier gas. LA-ICP-MS conditions were optimised on NIST 612 Trace Element in Glass CRM and the sample was ablated with a 50 μm spot size and a scan speed of 100 μm/s at a frequency of 20 Hz. The data were collated into a single image file using in-house developed software and visualised using FIJI. The limit of detection for mercury using LA-ICP-MS has been calculated to be 0.08 μg/g [23].

### Mercury content of the locus coeruleus

The human locus coeruleus in the rostral pons appears to be particularly susceptible to take up and retain circulating mercury [18] and has therefore been suggested to be an indicator of previous exposure to mercury [24]. The mercury content of the geniculate nuclei was therefore compared with that of paired locus coeruleus sections from the same individuals.

## Results

### Autometallography

AMG-detected mercury was present in the LGN or MGN of ten individuals in various combinations of endothelial cells, neurons, oligodendrocytes (**Table 1**) as well as in astrocytes.

**Table 1:** The distribution of mercury in cells of the lateral geniculate nucleus of ten mercury-positive individuals.

ID no.	Age group	Gender	Diagnosis	Cells			Other
				END	NEU	OLG	
G1	76–80	Female	Parkinson disease	+	++	+	
G2	36–40	Male	None	+	++	+	
G3	71–75	Female	Parkinson disease	+	++	+	
G4	36–40	Female	Bipolar disorder	++	++	+	
G5	71–75	Female	Multiple sclerosis	++	+	-	MGN
G6	61–65	Male	Depression	-	++	-	
G7	36–40	Male	None	++	-	+	MGN
G8	61–65	Male	Parkinson disease	+	+	+	
G9	96–100	Male	None	+	+	-	
G10	21–25	Male	Mercury injection	+	-	-	MGN

END: endothelial cell, MGN: same mercury distribution in medial geniculate nucleus, NEU: neuron, OLG: oligodendrocyte. Category of mercury distribution:–absent, + moderate, ++ marked.

#### Endothelial cells

**Fig 2:**
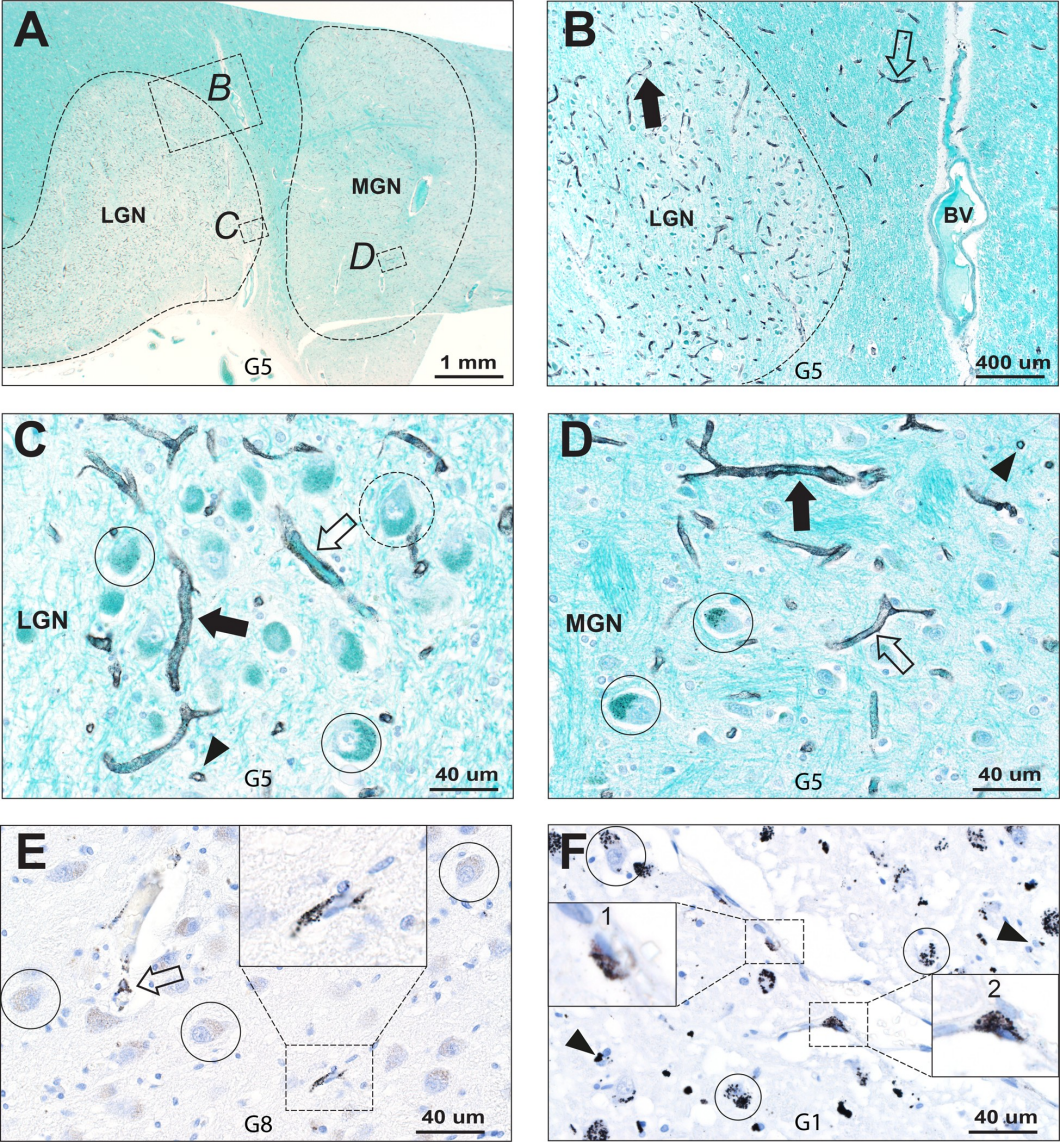
Mercury in geniculate blood vessels. (**A**) Low power view showing the anatomy of the LGN and MGN (within dashed lines). The boxes B, C and D are enlarged in following images. AMG/Luxol fast blue. (**B**) Most capillaries in the LGN contain mercury (eg, closed arrow), as do capillaries close to the LGN in the adjacent white matter (eg, open arrow). A nearby larger blood vessel (BV) in the white matter between the geniculate nuclei does not contain mercury. AMG/Luxol fast blue. (**C**) In the LGN, mercury is seen in endothelial cells of longitudinal (closed arrow) and transverse (arrowhead) capillary profiles. Two magnocellular neurons (circled) contain light particulate mercury, while one neuron (dashed circle) is mercury-free. AMG/Luxol fast blue. (**D**) In the MGN, mercury is seen in endothelial cells of longitudinal (closed arrow) and transverse (arrowhead) capillary profiles, similar to the LGN. Two magnocellular neurons (circled) contain moderate particulate mercury. AMG/Luxol fast blue. (**E**) Scattered LGN capillaries contain focal endothelial mercury (arrow and box). In this part of the LGN, neurons (eg, circled) do not contain mercury. AMG/hematoxylin. (**F**) Mercury is clustered around LGN endothelial cell nuclei (boxes 1 and 2). Nearby neurons (eg, circled) stain heavily for mercury, as do some oligodendrocytes (arrowheads). AMG/hematoxylin. G: identify number.

**Fig 3:**
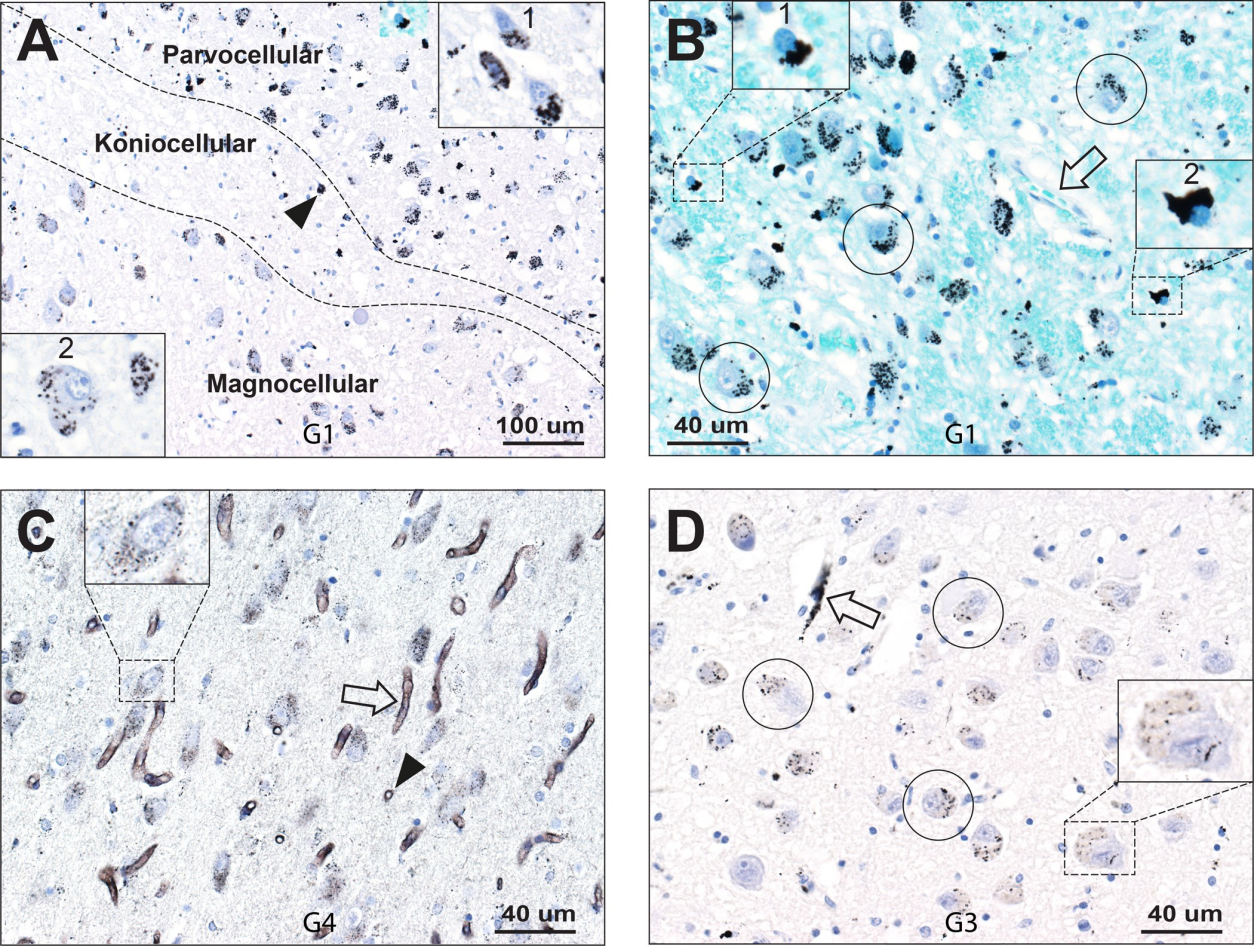
Mercury in geniculate neurons and oligodendrocytes. (**A**) Dense particulate mercury is seen in LGN parvocellular neurons (upper right, high power view in box 1). Magnocellular neurons (lower left, high power view in box 2) have less dense mercury on average than parvocellular neurons. A few small cells (eg, arrowhead) in the koniocellular layer contain mercury. AMG/hematoxylin. (**B**) Dense mercury deposits are seen in the shrunken cytoplasm of some oligodendrocyte cell bodies (eg, in boxes 1 and 2). Most neurons (eg, circled) contain mercury. One capillary (arrow) is mercury-free. AMG/Luxol fast blue. (**C**) Fine particulate mercury is seen in all LGN neurons (eg, box) and diffuse faint mercury staining is seen in the neuropil. Longitudinal (arrow) and transverse (arrowhead) capillary profiles show mercury in endothelial cells. AMG/hematoxylin. (**D**) Moderate scattered particulate mercury is seen, on a background of light yellow-brown lipofuscin, in many LGN neurons (eg, circled, and box). A capillary (arrow) has dense endothelial mercury. AMG/hematoxylin. G: identify number.

In nine of the mercury-affected geniculate nuclei, mercury was present in the endothelial cells of capillaries (**Table 1**). Capillaries containing mercury were situated within, and in some people also immediately adjacent to, the geniculate nuclei **(Fig 2)**. Mercury was present either diffusely or focally in the cytoplasm of endothelial cells, or as focal collections adjacent to endothelial cell nuclei (**Figs 2 and 3**). There was no consistent relationship between the amount of endothelial mercury and the mercury content of neurons or oligodendrocytes: widespread capillary mercury could be associated with little or no neuronal and oligodendrocyte mercury, and widespread neuronal mercury with little or no endothelial mercury (**Table 1 and Figs 2 and 3**).

#### Neurons

Mercury-containing LGN neurons were present in all magnocellular, parvocellular, and koniocellular layers. Parvocellular neurons appeared on average to have a greater mercury density than magnocellular neurons (**Fig 3**). In five people the great majority of LGN neurons contained mercury (**Table 1**). Mercury could be seen either as discrete small or medium-sized deposits in the cytoplasm, or as small grains on a background of lipofuscin (**Fig 3**).

#### Oligodendrocytes

**Fig 4:**
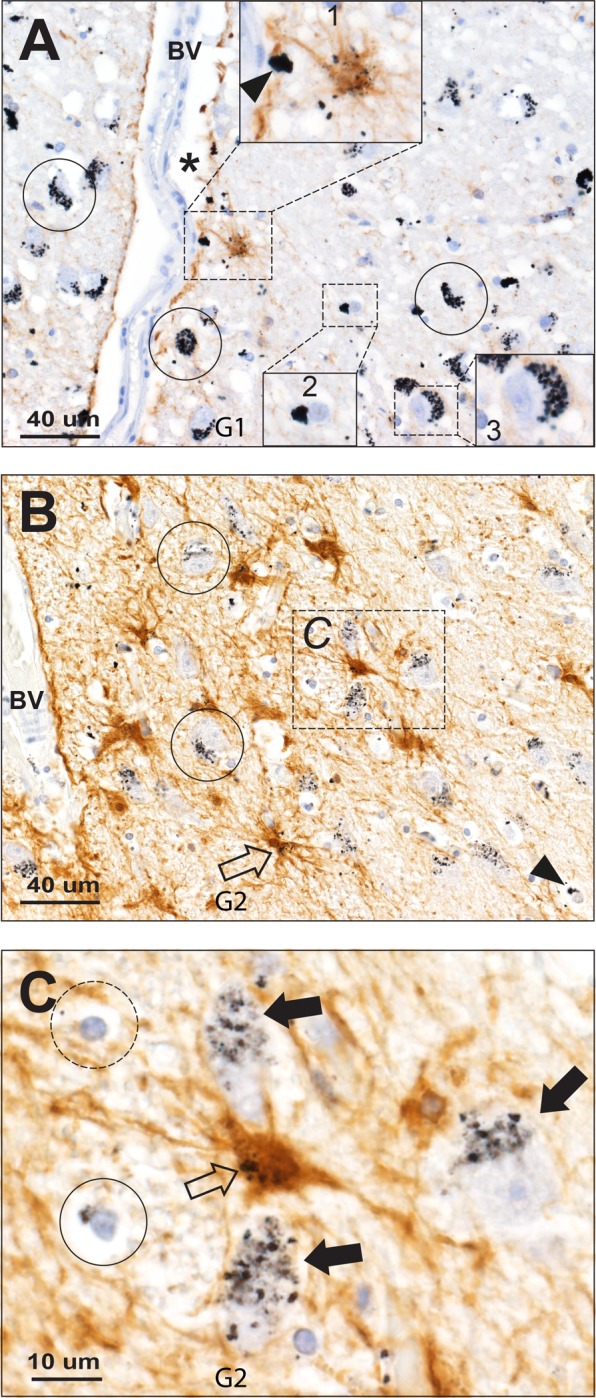
Mercury in geniculate astrocytes. (**A**) A brown-immunostained perivascular astrocyte in the LGN contains particulate mercury in its cytoplasm and processes (box 1). Particulate mercury (arrowhead) is present in the perivascular glia limitans, next to the artefactually-expanded perivascular space (*) of a small blood vessel (BV). Mercury is present adjacent to the nucleus of some oligodendrocytes (eg, box 2) and in the cytoplasm of neurons (eg, box 3). AMG/hematoxylin/GFAP. (**B**) Numerous perivascular brown-immunostained hyperplastic astrocytes in this LGN contain black mercury deposits (eg, arrow and box C, enlarged in 4C). Most neuronal (eg, circled) and some oligodendrocyte (arrowhead) cell bodies contain mercury. AMG/hematoxylin/GFAP. (**C**) Enlarged view of box C in (B) shows mercury in the cytoplasm of a hypertrophic astrocyte (open arrow) with processes extending to surrounding mercury-containing neurons (closed arrows) and oligodendrocytes, one with cytoplasmic mercury (circled) and one without (dash circled). AMG/hematoxylin/GFAP. G: identify number.

Oligodendrocytes were recognised by their characteristic small, round, densely hematoxylin-stained nucleus, with an artefactually shrunken cytoplasm. In six people mercury was readily detected in many (but not all) oligodendrocytes (**Table 1**). Mercury appeared as dense black AMG staining in the oligodendrocyte cytoplasm, adjacent to the nucleus (**Figs 2, 3 and 4**).

#### Astrocytes

Combined AMG and GFAP staining in two people showed a variation in astrocyte morphology and mercury content, even though both individuals had similar widespread mercury uptake in LGN capillaries, neurons and oligodendrocytes. In G1, mercury deposits were seen in a few normal-sized perivascular astrocytes, and in the perivascular glia limitans; in G2, on the other hand, numerous hypertrophic, predominantly perivascular, astrocytes contained mercury (**Fig 4**).

The distribution of mercury in endothelial cells, neurons, and oligodendrocytes in the three MGN was similar to that of the paired LGN from the same person. No cells in the adjacent hippocampus, or tail of the caudate nucleus, stained for mercury in any individual. Hematoxylin-only-stained sections showed no black deposits in any cells. Large amounts of lipofuscin were present in the geniculate neurons of all 50 individuals.

### Comparison of LGN and locus coeruleus mercury

All ten individuals with mercury-positive geniculate nuclei had more than 50% of locus coeruleus neurons that contained mercury. Of the 40 individuals in whom no geniculate mercury was found, numbers of mercury-containing locus coeruleus neurons were large (>50% of neurons) in 13, medium (11–50% of neurons) in 11, small (1–10% of neurons) in 3, and none in 13 people (**S1 Table**).

### LA-ICP-MS

**Fig 5:**
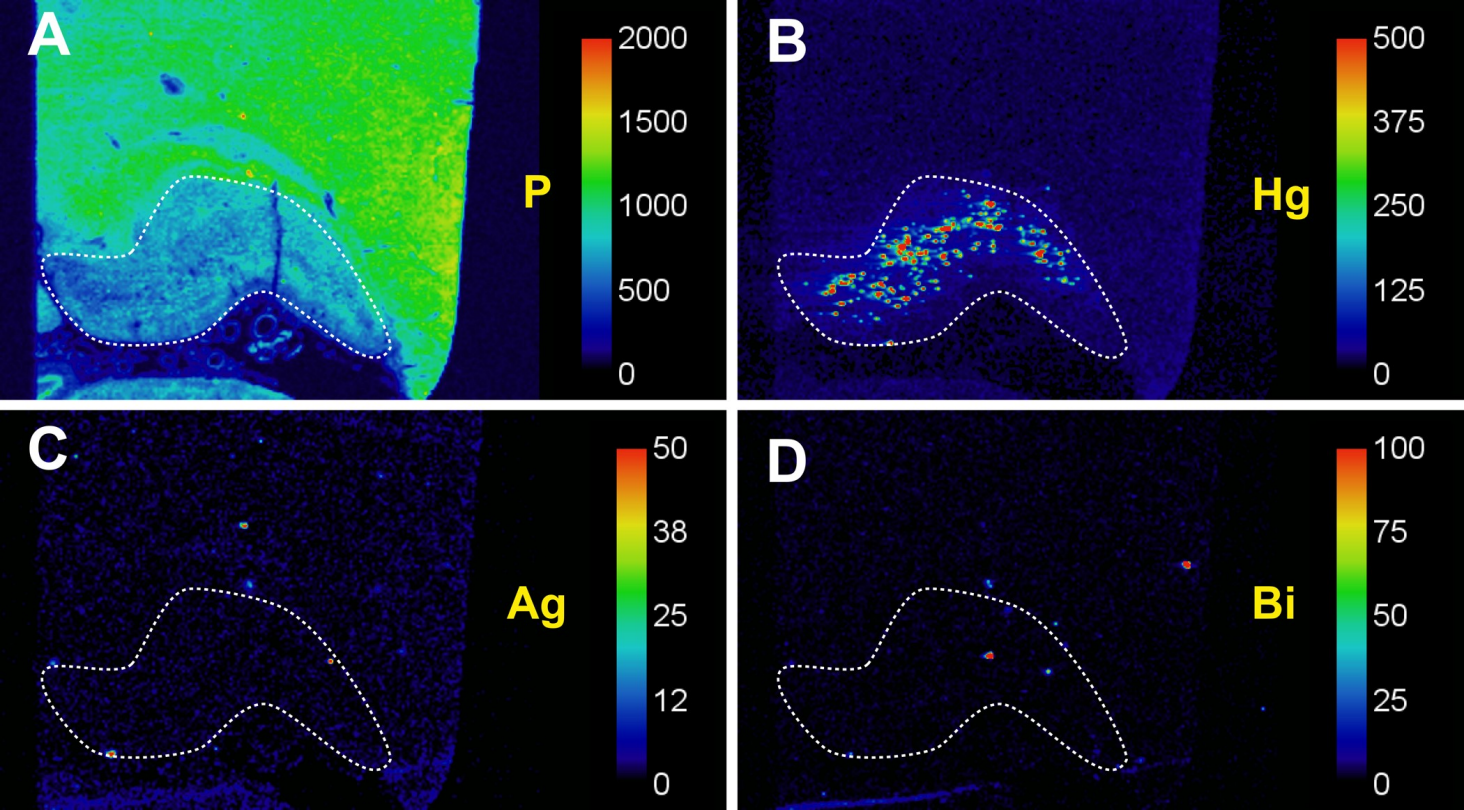
Laser ablation-inductively coupled plasma-mass spectrometry of an AMG-positive lateral geniculate nucleus (LGN, outlined). (**A**) Phosphorus scanning shows the phosphorus-rich cell layers of the LGN, compared to the uniformly dense phosphorus content of the adjacent tissue. (**B**) Mercury scanning shows a marked uptake of mercury in cells throughout most of the LGN. No significant amount of silver (**C**) or bismuth (**D**) is present in the LGN. Scale = counts per seconds (proportional to abundance).

AMG staining of the LGN in G1 was confirmed to be due to the presence of mercury on LA-ICP-MS, which showed no significant uptake of the other two metals that can be stained with AMG, silver and bismuth (**Fig 5**). No aluminium, cadmium, chromium, gold, iron, lead, or nickel was seen in the LGN.

## Discussion

Key findings of this study are that a proportion of people from a wide background of clinicopathological conditions had mercury in cells of their LGN and MGN. The distribution of mercury-containing cell types varied between individuals. These results raise the possibility that exposure to mercury, with preferential uptake in the geniculate nuclei, followed by inhibition of geniculate neuronal output and subsequent overactivity of the primary visual and auditory cortices, could be one factor underlying visual and auditory hallucinations.

**Fig 6:**
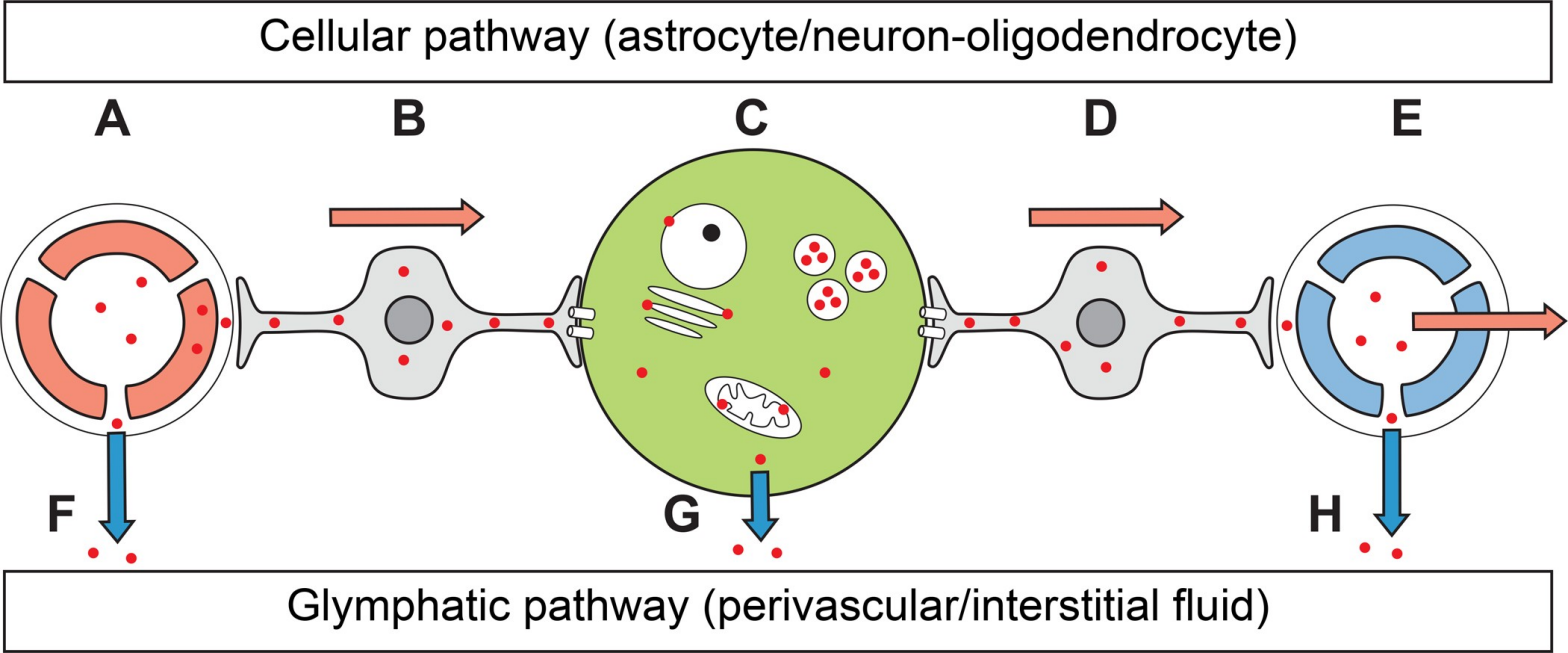
Potential pathways of mercury entry and exit in cells of the geniculate nuclei. (**A**) Circulating mercury (red dots) enters brain capillaries and passes into endothelial cells. Mercury passes through the perivascular space and is taken up by the endfeet of perivascular astrocytes. (**B**) Mercury collects in the cell bodies of perivascular astrocytes and is passed through gap junctions into neurons and oligodendrocytes. (**C**) Within neurons and oligodendrocytes, mercury is located within lysosomes, attached to mitochondria, endoplasmic reticulum, Golgi apparatus, and nuclear membranes, and free in the cytoplasm. (**D**) Mercury is removed from neurons through gap junctions into adjacent astrocytes, which transfer the mercury to venules. (**E**) Mercury enters the lumen of venules and is removed from the brain. Some mercury enters the perivenular space. (**F**) Some capillary mercury enters the interstitial fluid directly. (**G**) Some forms or mercury pass through the cell membrane directly into the interstitial space. (**H**) Interstitial mercury enters the perivenular space via the glymphatic pathway and exits the brain through the cerebrospinal fluid.

Mercury kinetics and metabolism within human cells is complex and only partially understood [25]. In the brain, methylmercury is slowly changed into inorganic mercury, the proximate toxic species. The effect of mercury within cells is likely to be a balance between the ingress of mercury from environmental exposure, the protective mechanisms within cells against mercury toxicity (such as binding to metallothioneins and selenium, and sequestration in lysosomes), and methods used to clear mercury from cells [26]. Most hallucinations are transitory, which may be the case because of this dynamic nature of toxic metal metabolism within cells. Mercury may be mostly toxic to cells while free and reactive in the cytoplasm early after exposure, when it is able to affect membranous organelles, and before it is ensconced within lysosomes or cleared from cells, either by a cellular pathway involving astrocytes [26], back through the blood-brain barrier [27], or via the glymphatic pathway that clears mercury from the interstitial fluid [28,29] (**Fig 6**).

Capillaries within, and immediately adjacent to, the geniculate nuclei, appeared to take up circulating mercury avidly, and this is probably the reason these geniculate cells were predisposed to take up mercury. Increased uptake of mercury by these endothelial cells may relate to the density or nature of mercury transporters in these cells [30–32]. Of note, all individuals with geniculate mercury also had many locus coeruleus neurons containing mercury. The locus coeruleus plays a role in maintaining the blood-brain barrier [33], so if the locus coeruleus neurons that contained damaging mercury were those supplying microvessels in the geniculate nuclei, circulating mercury could enter the geniculate nuclei readily via leaky capillaries.

Mercury within neurons varied from fine or dense particulate accumulations in the cell body, to scattered deposits within lipofuscin. Ultrastructural studies of neuronal mercury accumulation in experimental animals indicate that most mercury accumulates in lysosomes, some localises to membranous organelles such as mitochondria, the endoplasmic reticulum, the Golgi apparatus, and nuclear membranes, while some remains free in the cytoplasm [34–36]. The different patterns of mercury deposition seen in our geniculate neurons probably represent differences in the form of mercury exposed to (eg, vapor, organic, or inorganic), the frequency of exposure, and the time elapsed since last exposure. (**1**) The pattern of dense particulate neuronal mercury, probably in lysosomes (Fig 3B), is likely to represent continual or repeated exposures to mercury. Lysosomes play an important part in many cell processes [37], some of which could be affected by large amounts of mercury. A potential clinical correlation of this pattern of mercury deposition comes from individual G2, a fisherman who drowned at sea, who probably had a diet rich in fish, with large predatory fish having a high mercury content [38]. (**2**) The pattern of fine particulate neuronal mercury, as well as diffuse mercury in the neuropil (Fig 3C), is likely to result from recent exposure to mercury. Individual G4 died a few days after suffering extensive burns from ignited kerosene, which can contain mercury [39]. (**3**) Small scattered mercury particles on a background of lipofuscin (Fig 3D) probably indicate previous remote exposure to mercury, with partial removal of mercury from the cell body.

Our findings of mercury adjacent to the nuclei of oligodendrocytes recapitulates findings in the cerebral cortex from this man who injected himself with metallic mercury [40], as well as ultrastructural studies of rats exposed to mercury which showed mercury binding to oligodendrocyte nuclear membranes [34] and lysosomes [36]. Mercury in oligodendrocytes could affect myelin metabolism and axonal conductance, which has the potential to slow nerve impulses from the geniculate nuclei to their respective primary cortices.

Astrocytes are known depots for metals, and play a major part in the uptake, storage and release of toxic metals [26]. In two of our samples, in which LGN neurons and capillaries had similar amounts of mercury, astrocyte morphology differed markedly. This probably represents differences in the time between mercury exposure and tissue sampling, with the hyperplastic astrocytes reacting to recent exposure. Astrocytes are likely to play a role in both the accumulation and release and mercury [26], but since we could sample the geniculate nuclei at only one time point, we were unable to determine in which direction (from blood vessel to astrocyte, or from astrocyte to blood vessel) mercury in astrocytes was travelling.

Evidence from epidemiological and clinical studies supports the possibility that environmental toxicants such as mercury play a part in visual and auditory hallucinations. Exposure to environmental pollution, including heavy metals, has long been considered a possible risk factor for psychiatric disorders such as schizophrenia that are frequently accompanied by hallucinations [41–43]. Visual and auditory hallucinations often occur together, giving rise to the concept of ‘bound’ hallucinations [7]. The closely adjacent LGN and MGN nuclei in our study shared the same pattern of mercury accumulation which could explain why visual and auditory hallucinations so often co-exist. An alternative explanation, that the same pathology is affecting the far-apart primary visual and auditory cortices, seems less likely. Hallucinations are not uncommon in conditions in which we found geniculate mercury, namely Parkinson disease [7], depression [44], bipolar disorder [45] and multiple sclerosis [8]. Three of our mercury-affected geniculate individuals had no known major medical conditions, but visual hallucinations have been recorded in 7% of a general community [46].

Mercury exposure alone is not enough to result in mercury accumulation in the geniculate nuclei. For example, the individual who injected himself with metallic mercury, and who had large deposits of mercury in his body [40], had only minor geniculate endothelial mercury deposits, and none in his geniculate neurons or oligodendrocytes. Furthermore, while all individuals with geniculate mercury also had many locus coeruleus neurons containing mercury, a probable marker for previous mercury exposure [18], several people had locus coeruleus neurons containing mercury but no mercury in their geniculate nuclei. A possible explanation for the latter situation is that mercury had been cleared over time from their geniculate nuclei, but was retained in the locus coeruleus indefinitely because of its content of metal-binding neuromelanin [47,48].

It has long been noted that geniculate neurons contain a large amount of the ‘wear-and-tear’ pigment, lipofuscin [47,49–51], and this was the case in all our samples. The origin of lipofuscin remains obscure, but the large amount of lipofuscin that accumulates on aging in the geniculate neurons suggests these geniculate neurons are repeatedly exposed to toxicants that need to be processed by lysosomes [47]. Other neurons that have been shown to have a predilection to accumulate mercury, such as spinal [52] and cortical [53] motor neurons, also usually have large lipofuscin deposits. Lipofuscin may therefore turn out to be a marker of toxicant exposure to neurons. Despite the LGN showing marked lipofuscin accumulation during aging, LGN neurons appear to be resistant to age-related neuronal loss [51]. This may be because LGN neurons have efficient mechanisms of clearing toxicants such as mercury from their cell bodies, leaving behind lipofuscin as a signature of previous damage to organelles such as mitochondria [51].

People with schizophrenia have frequent visual and auditory hallucinations [1], but we found no mercury in the geniculate nuclei of the seven people with schizophrenia that we studied. This may be because mercury exposure occurred early in life and damaged the geniculate neurons, with the mercury being cleared from these neurons before death (a ‘hit and run’ situation). On the other hand, another toxicant currently not detectable at the cellular level could be causing geniculate neuronal damage. Further cell-based elemental analyses, such as synchrotron X-ray fluorescence microscopy, performed on geniculate cells could shed light on this issue, though this technique requires cryofixed sections of brain tissue for reliable results [54].

Neurons from the retina play only a small part in afferent stimuli to the LGN [55], and far from being only a visual relay station, multiple functions have now been ascribed to the LGN [55–57]. One possibility that has been raised is that damage to the LGN, by hindering light signals entering the suprachiasmatic nucleus, could lead to changes in circadian rhythms that cause sleep disturbances [58]. In addition, accumulation of bismuth has been described in the suprachiasmatic nuclei of mice [59], and bismuth affects the same neuronal types as mercury [13], but it is not known if mercury accumulates in the human suprachiasmatic nucleus. Sleep disturbances and hallucinations often co-exist in psychiatric conditions [60], so further investigations of toxicants in both the LGN and suprachiasmatic nucleus may be of value.

This study has several limitations. (**1**) We had access only to major clinical diagnoses of our study population, so we do not know whether any specific individual had experienced visual or auditory hallucinations. Ideally, imaging of metals within the geniculate nuclei, together with functional analyses of geniculate nuclei activity, would be undertaken at a time when hallucinations were being experienced, but this is not technically possible at present. (**2**) We do not know how and when our study population were exposed to mercury. In a future study, people who have frequent hallucinations could be asked to keep a detailed diary of dietary intake (especially of mercury-containing fish), bruxism or dental manipulations what might increase mercury uptake from silver amalgam dental restorations, and occupational activities that could expose them to mercury [38], to see if any mercury-related associations with the onset of hallucinations could be found. This could be expanded to population studies to see if the frequency of hallucinations increases in people exposed to mercury from small-scale gold mining [61], living near coal-fired power stations [61], volcanic activity [62], or wildfires [63–65]. (**3**) We did not have access to complete medical histories of these individuals, and so are not able to determine whether any medications taken by them could have altered mercury toxicokinetics. (**4**) Only a few MGN were available for study. This is because routine neuropathological sampling of the hippocampus usually includes the LGN, but seldom the more medially-situated MGN. Future prospective sampling of the MGN would be needed to comprehensively study toxicants in the MGN. (**5**) Autometallography can detect only the inorganic form of mercury, but since this is the proximate toxic form of the metal [25] for clinical purposes this is likely to be the most important form to detect. (**6**) Comparisons of our autometallography results with previous measurements of mercury in the geniculate nuclei and other parts of the brain are difficult, since we looked at mercury within individual cells, while the previous report of human geniculate mercury used bulk chemical analyses of fresh tissue [9]. We did not have access to fresh tissue to measure mercury content in the whole brain. (**7**) We were not able to quantitative the amount of mercury in individual cells using these methods. One way to do this is to use synchrotron x-ray fluorescence microanalysis, but this requires fresh frozen sections of tissue [54], which were not available to us. This issue warrants further study, since other metal toxicants such as lead have been suggested to be triggers for hallucinations [66].

In conclusion, this study indicates that a proportion of people from a mixed clinicopathological background have mercury in cells of their lateral and medial geniculate nuclei. This raises the possibility that this neurotoxicant affects the functions of these nuclei, which could precipitate the onset of visual and auditory hallucinations, because of subsequent overactivity of other afferents to the primary visual and auditory cortices. Although these findings do not provide a direct link between mercury in the geniculate nuclei and hallucinations, they suggest that further investigations into toxicant-induced hallucinations are warranted.

## Supporting information

S1 Table Clinicopathological details and cellular mercury in 50 individuals. (DOCX)
