# Supplementary material for: Elemental imaging shows mercury in cells of the human lateral and medial geniculate nuclei
Source: PLoS One. 2020 Apr 22;15(4):e0231870. doi: 10.1371/journal.pone.0231870 (PMC7176133; doi:10.1371/journal.pone.0231870)
Supplement: S1 Table — (DOCX) [file pone.0231870.s001.docx]

**Supplementary Table 1.** Clinicopathological details and cellular mercury in 50 individuals.

| **ID no.** | **Age range** | **Gender** | **Diagnosis** | **Cause of death** | **Mercury** | | |
| --- | --- | --- | --- | --- | --- | --- | --- |
|  |  |  |  |  | **LGN** | **MGN** | **LC** |
| G1 | 76-80 | Female | Parkinson disease | Cardiovascular | POS | NA | 3+ |
| G2 | 36-40 | Male | None | Drowning | POS | NA | 3+ |
| G3 | 71-75 | Female | Parkinson disease | Infection | POS | NA | 3+ |
| G4 | 36-40 | Female | Bipolar disorder | Burns | POS | NA | 3+ |
| G5 | 71-75 | Female | Multiple sclerosis | Infection | POS | POS | 3+ |
| G6 | 61-65 | Male | Depression | Suicide | POS | NA | 3+ |
| G7 | 36-40 | Male | None | Cardiovascular | POS | POS | 3+ |
| G8 | 61-65 | Male | Parkinson disease | Drowning | POS | NA | 3+ |
| G9 | 96-100 | Male | None | Trauma | POS | NA | 3+ |
| G10 | 21-25 | Male | Mercury injection | Suicide | POS | POS | 3+ |
| G11 | 81-85 | Female | Alzheimer disease | Infection | NEG | NA | 3+ |
| G12 | 91-95 | Female | Alzheimer disease | Infection | NEG | NA | 3+ |
| G13 | 86-90 | Female | Alzheimer disease | Trauma | NEG | NA | 2+ |
| G14 | 96-100 | Female | Alzheimer disease | Trauma | NEG | NA | 2+ |
| G15 | 86-90 | Female | Alzheimer disease | Cardiovascular | NEG | NA | 1+ |
| G16 | 36-40 | Female | Anorexia nervosa | Undernutrition | NEG | NEG | 3+ |
| G17 | 31-35 | Female | Bipolar disorder | Suicide | NEG | NA | 3+ |
| G18 | 46-50 | Female | Bipolar disorder | Drug overdose | NEG | NA | 2+ |
| G19 | 31-35 | Male | Depression | Trauma | NEG | NA | 3+ |
| G20 | 36-40 | Male | Depression | Suicide | NEG | NA | 1+ |
| G21 | 36-40 | Male | Depression | Suicide | NEG | NEG | 0+ |
| G22 | 31-35 | Female | Depression | Suicide | NEG | NA | 0+ |
| G23 | 26-30 | Male | Huntington disease | Suicide | NEG | NEG | 3+ |
| G24 | 56-60 | Male | Huntington disease | Undetermined | NEG | NA | 0+ |
| G25 | 96-100 | Female | Lewy body disease | Infection | NEG | NA | 0+ |
| G26 | 61-65 | Male | Multiple sclerosis | Undetermined | NEG | NA | 0+ |
| G27 | 61-65 | Female | Myotonic dystrophy | Cardiovascular | NEG | NA | 2+ |
| G28 | 101-105 | Female | None | Cardiovascular | NEG | NA | 3+ |
| G29 | 56-60 | Female | None | Drowning | NEG | NA | 3+ |
| G30 | 66-70 | Male | None | Drowning | NEG | NA | 3+ |
| G31 | 41-45 | Female | None | Suicide | NEG | NA | 3+ |
| G32 | 26-30 | Male | None | Suicide | NEG | NA | 2+ |
| G33 | 16-20 | Male | None | Suicide | NEG | NA | 0+ |
| G34 | 16-20 | Male | None | Suicide | NEG | NA | 0+ |
| G35 | 16-20 | Male | None | Suicide | NEG | NA | 0+ |
| G36 | 46-50 | Female | None | Trauma | NEG | NA | 0+ |
| G37 | 91-95 | Female | Parkinson disease | Cardiovascular | NEG | NA | 3+ |
| G38 | 66-70 | Male | Parkinson disease | Cardiovascular | NEG | NA | 2+ |
| G39 | 76-80 | Female | Parkinson disease | Cardiovascular | NEG | NA | 2+ |
| G40 | 76-80 | Male | Parkinson disease | Undetermined | NEG | NA | 1+ |
| G41 | 76-80 | Male | Parkinson disease | Infection | NEG | NA | 0+ |
| G42 | 76-80 | Female | Parkinson disease | Infection | NEG | NA | 0+ |
| G43 | 86-90 | Male | PSP | Cardiovascular | NEG | NA | 2+ |
| G44 | 36-40 | Male | Schizophrenia | Cardiovascular | NEG | NEG | 3+ |
| G45 | 26-30 | Male | Schizophrenia | Infection | NEG | NA | 3+ |
| G46 | 26-30 | Female | Schizophrenia | Suicide | NEG | NA | 2+ |
| G47 | 31-35 | Male | Schizophrenia | Trauma | NEG | NA | 2+ |
| G48 | 46-50 | Male | Schizophrenia | Undetermined | NEG | NA | 2+ |
| G49 | 26-30 | Male | Schizophrenia | Drug overdose | NEG | NA | 0+ |
| G50 | 41-45 | Female | Schizophrenia | Suicide | NEG | NA | 0+ |

ID: identity, LC: locus ceruleus, LGN: lateral geniculate nucleus, MGN: medial geniculate nucleus, NA: not available, NEG: negative, POS: positive, PSP: progressive supranuclear palsy. See text for grading of LC mercury.
